# Acrodermatitis Enteropathica in a Child in Bahrain: A Case Report and Literature Review

**DOI:** 10.7759/cureus.78545

**Published:** 2025-02-05

**Authors:** Hasan M Isa, Zainab H Ali, Kawthar M Abdulla, Zainab J Alshaikh, Maryam Y Busehail

**Affiliations:** 1 Department of Pediatrics, Arabian Gulf University, Manama, BHR; 2 Department of Pediatrics, Salmaniya Medical Complex, Manama, BHR

**Keywords:** acrodermatitis enteropathica, bahrain, deficiency, mutation, slc39a4 gene, zinc

## Abstract

Acrodermatitis enteropathica (AE) is a rare autosomal recessive disorder caused by a mutation in the zinc transporter gene, leading to impaired zinc absorption. A triad of periorificial dermatitis, alopecia, and diarrhea is the characteristic clinical presentation, although symptoms may vary with age. This disease typically manifests during infancy, particularly during the weaning process. The diagnosis can be confirmed through a thorough history, clinical findings, and laboratory investigations, mainly plasma zinc level assessment with or without genetic testing. Lifelong zinc supplementation is the standard treatment. This case report describes a 19-month-old Yemeni girl residing in Bahrain who was diagnosed with AE. The patient's main presentation was periorificial dermatitis involving the eyes, nose, mouth, ears, nape, and more extensively around the diaper area. The diagnosis was confirmed by clinical exome sequencing, which demonstrated a homozygous missense variant in exon 3 of the solute carrier family 39 member 4 (SLC39A4) gene. Accordingly, zinc replacement therapy was started, resulting in an improvement in the patient's condition. This case report highlights the clinical characteristics and genetic features of this disease.

## Introduction

Acrodermatitis enteropathica (AE) is a rare autosomal recessive disorder [1]. AE is caused by mutations in the zinc transporter gene named solute carrier family 39 member 4 (SLC39A4) [2]. Pathogenic mutations were either homozygous or compound heterozygous in most patients [2]. These mutations affect zinc absorption, leading to zinc deficiency [1]. Brandt first described AE in 1936 [3]. Thereafter, Danbolt and Closs recognized it as a separate illness in 1942 [4]. AE has an estimated incidence of one per 500,000 children worldwide [5].

AE typically appears in infancy, during the weaning process for breast milk-fed infants, and earlier in formula-fed infants [5]. AE can be found in any ethnic group, regardless of sex [1]. Periorificial dermatitis, alopecia, and diarrhea are the classic disease triad [2]. Skin rash can present as eczematous pink scaly plaques, which may develop into vesicular, bullous, or desquamative lesions [5]. However, if not treated, it can cause erosions and may become secondarily infected with bacteria and *Candida albicans* [5]. Moreover, angular cheilitis is a common early symptom followed by paronychia [5]. Patients with advanced disease may also have growth delay, mental delay, poor wound healing, anemia, photophobia, hypogeusia, anorexia, delayed puberty, and hypogonadism in males [5].

The diagnosis of AE is based on a thorough history and clinical findings, in addition to laboratory investigations showing low plasma zinc levels [6]. However, many cases diagnosed with AE had normal zinc levels [6,7]. Furthermore, a decreased serum or plasma zinc level does not necessarily signify zinc deficiency [5]. Therefore, genetic testing for SLC39A4 mutations is the definitive diagnostic test for AE [8].

Zinc replacement therapy (3 mg/kg/day) should be initiated once AE diagnosis is confirmed [5]. Rapid improvement in clinical condition within days to weeks is considered a typical response to zinc supplementation and supports the diagnosis [5,6]. Yet, zinc dosage can be adjusted to higher or lower than 3 mg/kg/day to normalize the patient's zinc level [5]. Serum or plasma zinc levels should be checked every three to six months after zinc supplementation starts [5]. Although AE is a rare disease, several cases have been reported in the Gulf region and worldwide [6-12]. However, this disease was not previously reported in the Kingdom of Bahrain. Accordingly, this article presents a report on the first case of a child diagnosed with AE in Bahrain and a literature review.

## Case presentation

**Figure 1:**
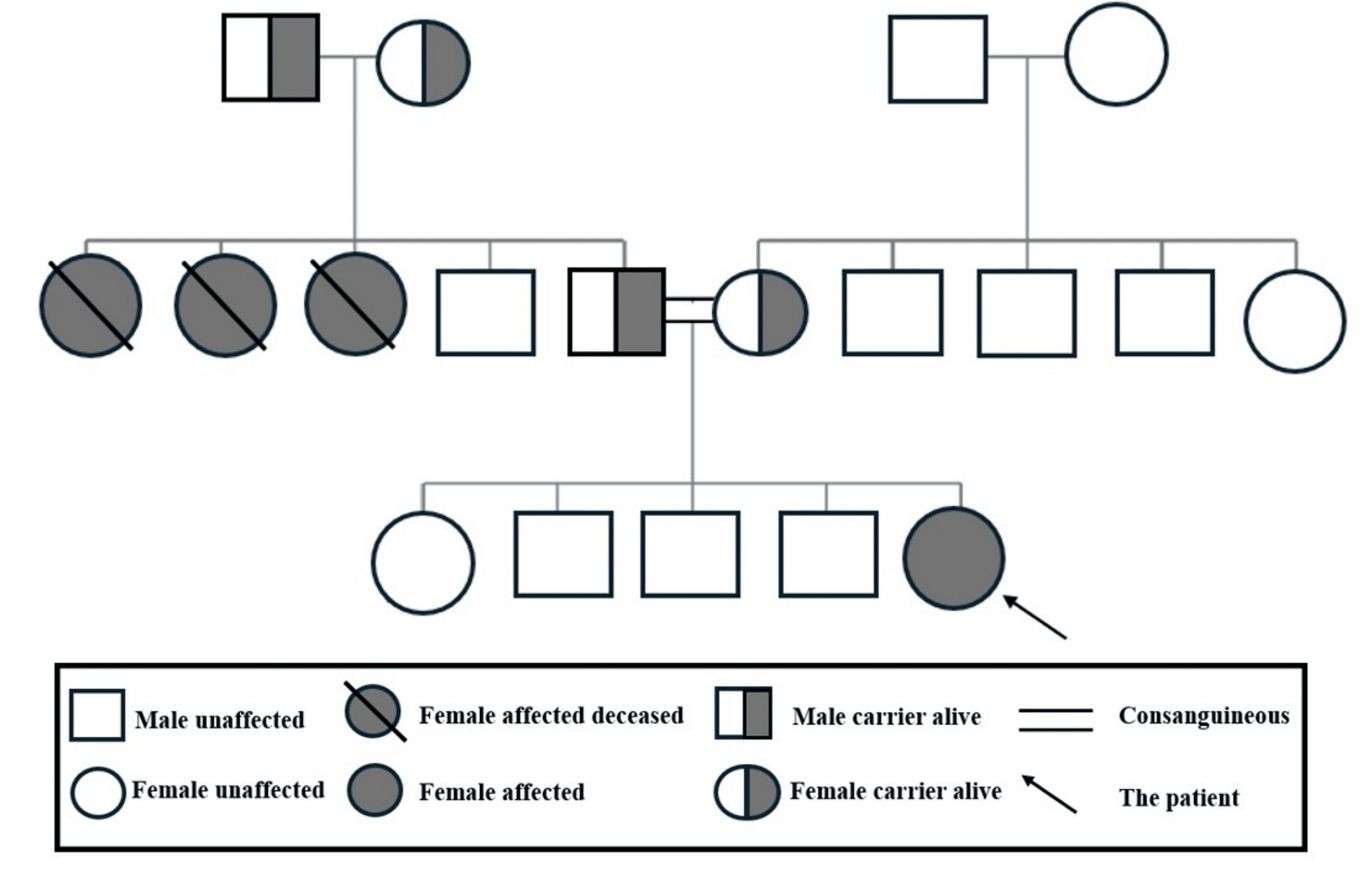
Family pedigree of the 19-month-old child with acrodermatitis enteropathica. Image credits: Hasan M. Isa, Zainab H. Ali, Kawthar M. Abdulla

This is the case of a 19-month-old Yemeni girl who was born at 39 weeks via normal vaginal delivery. The patient is the fifth child of apparently healthy consanguineous parents who are third-degree relatives. She has a positive family history of zinc deficiency in three of her aunts. Two of them passed away at the ages of four and five as they were not treated. The third one was on zinc supplementation; however, she also passed away by the age of 20 due to renal failure (Figure 1).

**Figure 2:**
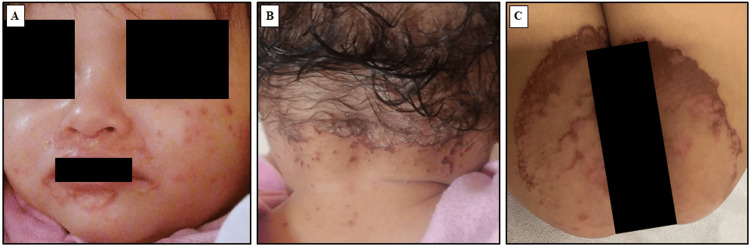
The child with acrodermatitis enteropathica. (A) Periorificial skin lesions; (B) Lesions at the nape; (C) Lesions at the napkin area.

The patient was asymptomatic on exclusive breastfeeding until the age of seven months when she started to develop a skin rash of two weeks duration. The rash was itchy, red, and scaly with pustules and was located mainly around the eyes, nose, mouth, ears, nape, and more extensively around the diaper area (Figure 2).

The patient was brought to the accident and emergency department where she was investigated. Anemia, leukocytosis, thrombocytosis, low neutrophil percentage, high lymphocyte percentage, and low alkaline phosphatase (ALP) were detected, while the results of complete blood count, liver function tests, and renal function tests were otherwise unremarkable (Table 1).

**Table 1:** Laboratory results of the child with acrodermatitis enteropathica. Data are presented as numbers or percentages. RBCs, red blood cells; MCV, mean cell volume; MCH, mean cell hemoglobin; NR, no record; WBCs, white blood cells

Laboratory test	Normal range	Patient age (months)					
		7	8	9	11	16	19
Hemoglobin (g/dL)	12-14.5	9.5	9.8	10.1	11.1	11.6	NR
Hematocrit (%)	33-45	29.5	31.3	31.8	35.4	35.5	NR
MCV (fL)	80-97	78.2	79.8	79.3	78.3	82.4	NR
MCH (pg)	27-33	25.2	25.0	25.2	24.6	26.9	NR
RBCs (×10^12^/L)	3.9-5.2	3.8	3.9	4.0	4.5	4.3	NR
Platelets (×10^9^/L)	150-400	667	265	499	387	268	NR
WBCs (×10^9^/L)	3.6-9.6	14.9	9.2	18.8	17.0	13.5	NR
Neutrophils (%)	42.2-75.2	25.8	NR	26.8	6.2	22.5	NR
Lymphocytes (%)	20.5-51.1	65.0	77.4	60.0	81.0	60.0	NR
Iron (µmol/L)	9.0-30.4	NR	NR	7.0	6.4	NR	8.5
Transferrin (g/dL)	2.5-3.8	NR	NR	2.0	2.2	NR	2.4
Transferrin saturation (%)	15-33	NR	NR	14	11	NR	14
Zinc (µmol/L)	7.7-18.4	NR	9.6	NR	8.2	NR	11.7
Vitamin D (nmol/L)	≥50	NR	NR	NR	NR	NR	27
Alkaline phosphatase (U/L)	120-450	107	90	NR	NR	130	154
Total protein (g/L)	57-82	60	60	NR	NR	65	NR
Albumin (g/L)	38-54	40	41	NR	NR	44	NR
Globulin (g/L)	15-30	20	19	NR	NR	21	NR
Total bilirubin (µmol/L)	5-21	4	3	NR	NR	4	NR
Alanine aminotransferase (U/L)	≤33	15	20	NR	NR	15	NR
G-glutamyl transferase (U/L)	≤38	15	17	NR	NR	15	NR

The patient’s management included intravenous fluid, dexamethasone, and analgesia. The itchiness improved, and she was discharged home on antihistamine syrup, neomycin sulfate-bacitracin ointment, beta-sitosterol ointment, and dexapanthenol cream to manage the rash.

At the age of eight months, the patient presented again to the accident and emergency department as her rash did not disappear. The patient was still receiving breast milk, but a standard milk formula was recently introduced. She was assessed by the dermatology team and was found to be stable apart from the previous skin rash, which further extended to her labia majora along with the development of paronychia. Thus, the clinical impression was most likely AE. Accordingly, routine laboratory tests were requested in addition to serum zinc and ALP levels (Table 1). The patient was started on zinc gluconate of 20 mg daily, zinc oxide topical cream, and an Aquaphor® healing ointment (Beiersdorf Inc., Germany). One week after starting the zinc supplementation, the requested zinc and ALP tests revealed a serum zinc level of 9.6 μmol/L, being at the lower normal limit (normal range: 7.7-18.4 μmol/L). The ALP level was 90 U/L (normal range: 120-450 U/L). The patient's rash subsided since then. Consequently, genetic testing was sent to confirm the diagnosis. In the genetics outpatient clinic, the parents were offered genetic testing and counseling regarding the risk of future pregnancies.

Thereafter, the patient presented to the primary health care several times due to recurrent upper respiratory tract infections. Routine laboratory tests were requested on three visits at nine, 11, and 16 months (Table 1). At the age of 16 months, the patient was referred from the primary health care center to the pediatrics rheumatology clinic due to broken fingernails. She was not compliant with zinc supplementation; however, the nails regrew normally after complete adherence. 

At the age of 18 months, genetic testing showed a homozygous missense variant in exon 3 of the SLC39A4 gene (chr8:g.144415295G<A; Depth: 154x) that resulted in an amino acid substitution of leucine for proline at codon 200 (p.Pro200Leu; ENST00000301305.8), confirming the diagnosis of AE.

**Figure 3:**
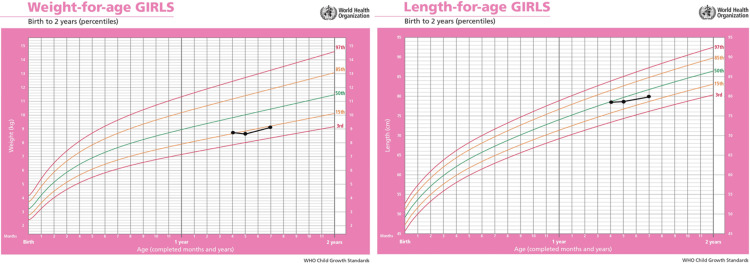
Growth charts of the child with acrodermatitis enteropathica. World Health Organization growth charts for both weight and height representing lower normal limits for age and sex.

At the age of 19 months, the patient was seen in the pediatrics gastroenterology clinic due to slow weight gain (Figure 3).

Dietary advice was given to the parents, and they were informed to proceed with zinc replacement therapy. A follow-up zinc level, ALP, iron profile, vitamin D level, celiac screening, and stool microscopy were requested. The results showed a normal zinc level of 11.7 μmol/L (normal range: 7.7-18.4 μmol/L), a normal ALP level of 154 U/L (normal range: 120-450 U/L), a low iron level of 8.5 µmol/L (normal range: 9.0-30.4 µmol/L), a low vitamin D level of 27 nmol/L (normal range: ≥50 nmol/L), and negative celiac serology, whereas the stool microscopy was unremarkable. The patient was supplemented with oral vitamin D drops and iron syrup.

## Discussion

AE is a rare autosomal recessive disorder caused by mutations in the zinc transporter gene SLC39A4 [1,2]. To the best of our knowledge, this report presents the first case of a child with AE in Bahrain, contributing to the global incidence of this disease [5]. Regional reports have emerged primarily from areas with higher consanguinity rates, such as the Middle East [13]. Consanguineous marriages increase the prevalence of autosomal recessive genetic disorders [13]. Aside from our case, four patients with AE were previously reported from the Gulf region [6,8-10]. Al Naamani et al. [8] reported one case from Oman, in addition to four cases reported from Saudi Arabia by Alwadany et al. [9], Alsulami et al. [10], and Al Rashed et al. [6] who reported twins (Table 2).

**Table 2:** A literature summary of patients with acrodermatitis enteropathica. * indicates the present case. F, female; M, male; NR, not recorded; ALP, alkaline phosphatase; SLC39A4, solute carrier family 39 member 4

Authors	Year	Country	Sex	Presentation age	First presentation	Other presentations	Consanguinity	Family history	Serum zinc level	ALP level	Genetics	Zinc supplementation
Isa et al.*	2025	Bahrain	F	7 m	Rash (periorificial and napkin area)	Paronychia, brittle nails	Positive	Positive	Normal (after treatment)	Low	Homozygous missense variant in the SLC39A4 gene	Zinc gluconate (20 mg/kg/day)
Al Rashed et al. [6]	2016	Saudi Arabia	F	4 m (twins A and B)	Rash (perioral, perianal areas, hands, and feet)	Intermittent diarrhea, alopecia	Negative	Negative	Low	Normal	NR	Zinc supplementation (1 mg/kg/day)
Hua et al. [7]	2022	China	F	9 y	Rash (perioral, perianal, and acromelic areas)	Alopecia	Negative	Negative	Low	Normal	Novel heterozygous mutation in the SLC39A4 gene	Zinc sulfate (3 mg/kg/day)
Al Naamani et al. [8]	2019	Oman	F	2 m	Rash (perioral, hands, and napkin areas)	Alopecia, diarrhea, cracked lips	NR	NR	Low	Low	Not done	Elemental zinc (3 mg/kg/day)
Alwadany et al. [9]	2023	Saudi Arabia	M	10 y	Rash (hands and elbows), diarrhea, and abdominal pain	Poor appetite	Positive	Negative	Low	Normal	Done (not specified)	Zinc sulfate (10 mg/kg/day)
Alsulami et al. [10]	2024	Saudi Arabia	F	2 y	Psoriasiform-eczematous skin lesions (extremities)	Mild angular cheilitis, scaly patches on the vulva	Negative	Negative	Low	Normal	NR	Zinc sulfate (13 mg/kg)
Cleminson et al. [11]	2020	Canada	M	10 m	Red-to-brown plaques (knees, cheeks, and chin) and blistering and crust (hands and feet)	Nil	NR	Negative	Low	Low	Pathogenic variant of the SLC39A4 gene	Zinc gluconate (3 mg/kg)
Nistor et al. [12]	2016	Romania	M	14 m	Pustular lesions (periorificial, scalp inguinal, perianal, and thighs)	Alopecia, paronychia, psychomotor agitation, fall of eyebrows and eyelashes	NR	Positive	Low	Normal	Not done	Elemental zinc (3 mg/kg/day)

Consanguinity and family history are pivotal in diagnosing AE [1]. In this report, our patient is the fifth child of consanguineous parents with a family history of zinc deficiency in three of her aunts. Consanguinity was also reported by Alwadany et al. [9], while a positive family history was reported by Nistor et al. [12]. In contrast, no consanguinity or family history was noted in the other reviewed cases [6-8,10,11], reflecting a broad genetic diversity of AE even outside high-risk populations.

AE typically manifests during infancy or early childhood [5]. Particularly, it appears after weaning, when the zinc supply from breast milk diminishes [5]. Our patient presented at the age of seven months. Comparably, the age at presentation reported by Cleminson et al. [11] and Nistor et al. [12] was 10 and 14 months, respectively. In contrast, cases reported by Hua et al. [7] and Alwadany et al. [9] were presented at an older age (nine and 10 years, respectively). However, Al Rashed et al. [6] reported Saudi twins presented at four months, illustrating variations that were influenced by nutritional transitions and genetic predispositions [5,6]. Moreover, Al Naamani et al. [8] reported a case from Oman involving a premature infant who exhibited symptoms as early as two months, reflecting the increased vulnerability of preterm infants to zinc deficiency [5]. The clinical presentation of AE consists of a spectrum of symptoms and signs that includes the classic triad of dermatitis, alopecia, and diarrhea, along with systemic signs like behavioral changes, growth delay, and recurrent infections [2]. The classic triad was presented in patients reported by Al Naamani et al. [8] and Al Rashed et al. [6]. In this report, the patient's initial presentation was erythematous, itchy, and scaly rashes around the eyes, nose, mouth, ears, nape, and napkin area, which is consistent with the dermatological features of AE [14], despite the incomplete classic triad. Cleminson et al. [11] reported a case mimicking psoriasis with psoriasiform plaques, while the cases reported by Nistor et al. [12] and Hua et al. [7] were complicated by bacterial superinfections, highlighting the effect of secondary complications on the clinical picture and diagnosis. Comparatively, a 10-year-old Saudi patient reported by Alwadany et al. [9] had skin rash along with diarrhea and abdominal pain, which were both lacking in our patient. Moreover, our patient also had paronychia. Similarly, Nistor et al. [12] reported paronychia in their case in addition to psychomotor agitation. These findings underscore the variability in AE presentations from the classic triad to atypical symptoms, which may complicate diagnosis without a high index of suspicion.

The diagnosis of AE is based on clinical presentation, laboratory findings, and genetic testing [6]. Besides the typical presentation in infancy after weaning and the characteristic triad of symptoms [5,14], low plasma zinc levels can be helpful in AE diagnosis [5]. However, the cut-off values of the lower limits are not well defined [5]. Despite tissue depletion, normal zinc levels can also occur [6]. In addition, ALP (a zinc-dependent enzyme) is a helpful marker in the AE diagnosis [5,11]. Zinc deficiency also affects immune function, impairing neutrophil activity [15]. In this report, although the patient’s serum zinc level was checked after providing zinc supplements, the zinc level was near the lower limit of normal (9.6 mmol/L, normal range: 7.7-18.4 mmol/L), ALP was low (107 IU/L, normal range: 120-450 IU/L), and the neutrophils percentage was also low (25.8%, normal range: 42.2-75.2%). Zinc deficiency and variable ALP levels were reported across all cases reviewed in this article [6-12]. Al Rashed et al. [6] and Alsulami et al. [10] reported low zinc levels and normal ALP levels. However, Cleminson et al. [11] recorded low zinc (2.6 μmol/L) and low ALP (58 U/L) levels. Nistor et al. [12] also noted low zinc levels. This comparison highlights the variability of these markers.

Biopsies from skin lesions can be an additional diagnostic procedure [14]. Histopathological features of AE, such as parakeratosis and psoriasiform hyperplasia, are similar to those of other deficiency dermatitis like niacin or vitamin B3 deficiencies but with minimal differences [5]. A biopsy was performed in Alsulami et al.’s case [10] that revealed non-specific changes, and the patient was misdiagnosed with psoriasis. A biopsy was also conducted in Nistor et al.’s case [12], and it showed epidermal hyperplasia with acanthosis, clustered necrotic keratinocytes, parakeratosis, crusts, and intraepidermal vacuolization. Nonetheless, laboratory tests and skin biopsies might be helpful in the diagnosis, but they do not always lead to a definitive diagnosis [5].

AE results from mutations in the zinc transport gene and genetic testing confirms the diagnosis by identifying SLC39A4 mutations [2]. In this report, genetic testing revealed a homozygous missense mutation, c.599C>T, p.(Pro200Leu), in the SLC39A4 gene, aligning with findings reported by Cleminson et al. [11], where genetic studies revealed a similar mutation. Hua et al. [7] also identified the SLC39A4 gene mutation, but the variant was not specified. These findings emphasize the diagnostic utility of genetic testing, particularly in atypical cases or when clinical and laboratory findings are inconclusive [1]. Genetic testing was unavailable in some cases, such as Al Naamani et al. [8], due to logistical reasons.

The management of AE depends on lifelong zinc supplementation [14]. Rapid symptom resolution after zinc supplementation is considered diagnostic for AE [14]. In our case, zinc supplementation led to a rapid improvement in symptoms. However, non-compliance caused nail changes, which improved once therapy was resumed. Similarly, Al Naamani et al. [8] and Al Rashed et al. [6] showed rapid improvement in four and two weeks, respectively, after early treatment and adherence. Hua et al. [7] also demonstrated betterment in the patient's condition even though the rash was superinfected. Therefore, early diagnosis and adherence to therapy are critical to achieving favorable long-term outcomes [1].

## Conclusions

AE is an autosomal recessive disorder characterized by a triad of periorificial dermatitis, alopecia, and diarrhea. In this article, we report the first case of a child in Bahrain with AE, with a positive family history. The initial presentation was periorificial dermatitis, and multiple skin lesions, mainly in the napkin area. The diagnosis was confirmed by genetic testing, and she was treated with zinc supplementation and showed rapid improvement. Although AE is a rare disorder, it should be considered in the differential diagnosis of infants and children presenting with characteristic skin lesions, especially in the context of positive family history and consanguinity. Clinical observation, careful history taking, appropriate investigations, and timely zinc supplementation remain the cornerstones of effective management. Further research, including larger epidemiological studies, is needed to understand the disease burden and the genetic variations to optimize management strategies. Moreover, follow-up studies are required to assess the long-term effect of zinc therapy and identify potential complications.
